# The Role of Inhibition of Apoptosis in Acute Leukemias and Myelodysplastic Syndrome

**DOI:** 10.3389/fonc.2019.00192

**Published:** 2019-03-27

**Authors:** Amanda McBride, Sarah Houtmann, Lindsay Wilde, Carlos Vigil, Christine M. Eischen, Margaret Kasner, Neil Palmisiano

**Affiliations:** ^1^Department of Medical Oncology, Sidney Kimmel Cancer Center, Thomas Jefferson University, Philadelphia, PA, United States; ^2^Department of Medicine, Thomas Jefferson University, Philadelphia, PA, United States; ^3^Division of Hematology, Oncology and Blood and Marrow Transplantation, Department of Internal Medicine, University of Iowa, Iowa City, IA, United States; ^4^Department of Cancer Biology, Sidney Kimmel Cancer Center, Thomas Jefferson University, Philadelphia, PA, United States

**Keywords:** BCL-2, venetoclax, AML, ALL, MDS, intrinsic apoptosis

## Abstract

Avoidance of apoptosis is a key mechanism that malignancies, including acute leukemias and MDS, utilize in order to proliferate and resist chemotherapy. Recently, venetoclax, an inhibitor of the anti-apoptotic protein BCL-2, has been approved for the treatment of upfront AML in an unfit, elderly population. This paper reviews the pre-clinical and clinical data for apoptosis inhibitors currently in development for the treatment of AML, ALL, and MDS.

## Introduction

One of the well-described hallmarks of cancer is the ability to evade apoptosis. Upstream regulators and downstream effectors of apoptosis carefully regulate the precise dismantling of a cell in response to a wide variety of internal and external stimuli. At any point along the pathway malignancies can exploit these interactions for survival. Research convincingly demonstrates that acute leukemias and myelodysplastic syndrome (MDS) co-opt these mechanisms for both their development as well as resistance to traditional chemotherapies. Recently, many new drugs that work to promote apoptosis have been developed and studied. The B cell lymphoma-2 (Bcl-2) protein family includes key regulator of apoptosis, including the anti-apoptotic protein BCL-2. Venetoclax, an oral BCL-2 inhibitor and the prototype for this class of drugs, has shown promising efficacy in the treatment of several hematologic malignancies. First approved in 2016 for the treatment of relapsed/refractory CLL, venetoclax has now also been recently FDA approved for the treatment of acute myeloid leukemia (AML). However, to date, none of these drugs have been approved in acute lymphoblastic leukemia (ALL) or MDS. This review seeks to explain the role of the intrinsic pathway of apoptosis and the data for drugs targeting the Bcl-2 superfamily in the treatment of acute leukemias and MDS.

## Intrinsic Apoptosis and the BCL-2 Superfamily

Apoptosis is governed by two intertwined pathways that ultimately lead to cell death. The extrinsic pathway is activated in response to external signaling proteins, like tumor necrosis factor alpha (TNF-a) and FAS-L, which generate an intracellular signal through transmembrane receptor proteins. These signals in turn lead to the activation of caspases that are ultimately responsible for degradation of essential cellular proteins [see (1, 2) for a review of the extrinsic pathway].

**Figure 1:**
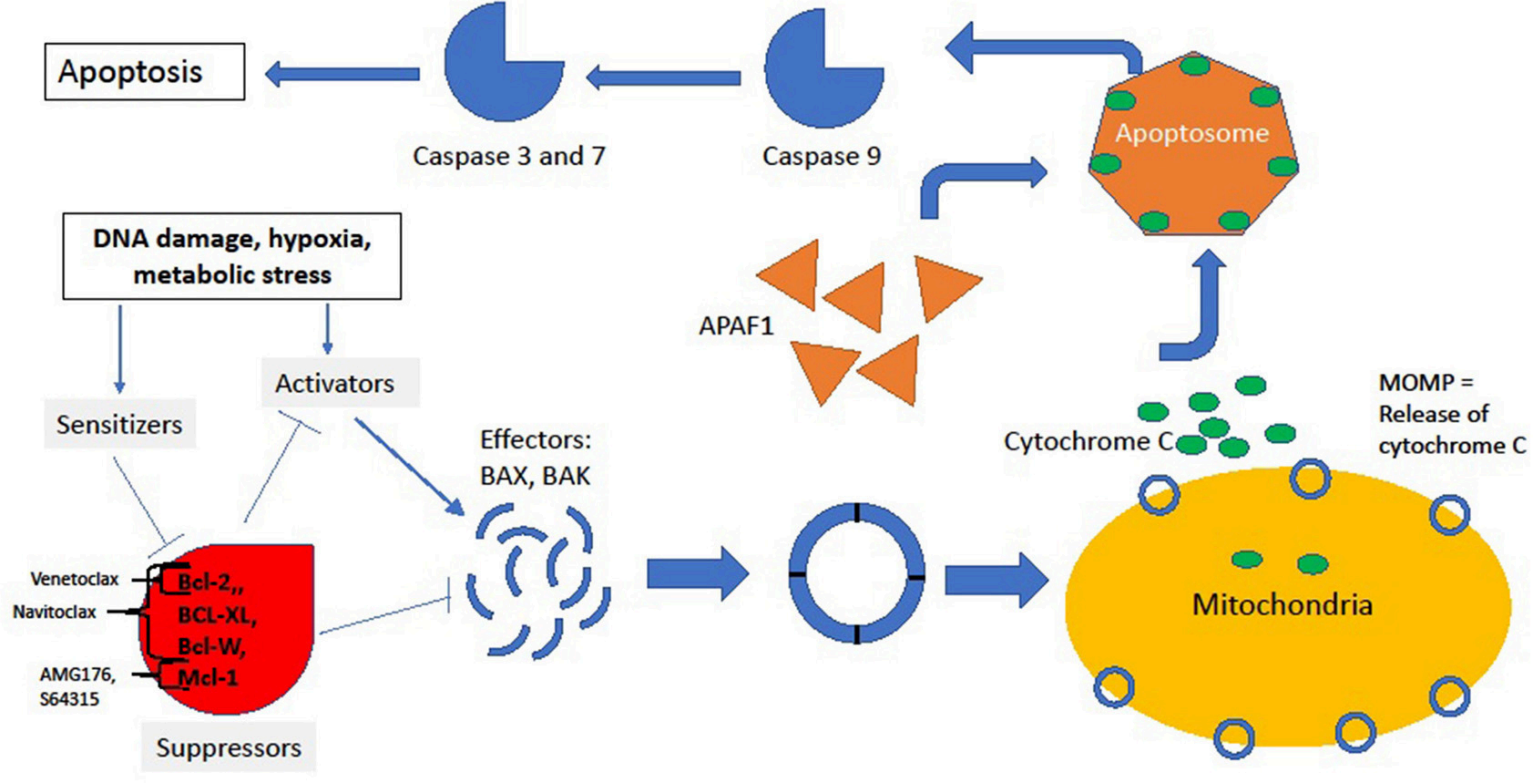
Intrinsic pathway. In the resting state, suppressors BCL-2, MCL1, BCL-XL, and BCL-W bind to and inhibit effectors and activators, thus preventing downhill cascade leading to apoptosis. The site of inhibition of key BCL-2 family inhibitors is noted by brackets. In response to stress, sensitizers bind to the suppressors, releasing the inhibitory effect of suppressors on effectors and activators. Thus, conformational change and oligmerization ensues with subsequent MOMP formation and release of cycotochrome C. Cytochrome C and Apaf-1 oligmerize into the apoptosome, leading to activation of caspases and, finally, cell death.

The intrinsic pathway is tightly regulated by the Bcl-2 superfamily of proteins. Following activation of the intrinsic pathway, a complex interplay between various members of the Bcl-2 family of proteins culminates in the release of cytochrome-C from “stressed” cells' mitochondria by the creation of pores in the mitochondrial outer membrane, a process denoted as mitochondrial outer membrane permeabilization (MOMP). The supergroup members share BCL-2-like homology domains 1-4 (BH1-BH4) and can be divided into various classes based on the impact of the protein on the apoptotic outcome of the cell: sensitizers, activators, suppressors and effectors. For a cell in resting state, suppressors of apoptosis (BCL-2, BCL-XL, MCL1, BCL-W among others) bind to effectors (BAX or BAK) and activators (BID and BIM) and suppress their activity. Conversely, as a result of upstream cell damage signaling (see Figure 1), the production of sensitizers and activators is induced. Sensitizers bind directly to anti-apoptotic proteins which causes their disassociation from effector and activator proteins. In turn, activators bind to effectors, inducing a conformational change in the effectors that allows for their oligomerization. This oligomerization event allows for the creation of pores in mitochondria that generates MOMP and induces the release of cytochrome-C through the mitochondrial membrane. Extra-mitochondrial cytochrome-C complexes with Apaf-1 which subsequently oligomerizes into a heptamer known as the apoptosome and recruits and activates caspase 9. Activated caspase 9 plays a central role in cleaving other effector caspases, and this ultimately leads to the proteolytic dismantling of the cell (3, 4).

Upstream of the intrinsic pathway, the central tumor suppressor p53 helps to orchestrate the cellular signals leading to apoptosis. In p53-competent cells, DNA damage initiates a broad cascade of responses, including the dissociation of p53 from its inhibitor MDM2 and its rapid translocation to the mitochondria. Once there, p53, through complex interactions, is thought to release pro-apoptotic Bax and Bak from inhibitory proteins like BCL-2 and BCL-XL, which functionally sequesters them and pushes the “stressed” cell toward apoptosis. Additionally, p53, when liberated from MDM2, acts as a transcription factor for BAX itself as well as for the initiator proteins PUMA and NOXA. PUMA and NOXA bind to BCL-XL, allowing for the release of Bax to form its effector oligomers. In certain cells, p53 may also transcriptionally repress BCL-2 (5). The sum of the effects of p53 activation are pro-apoptotic and, in part through its interactions with the Bcl-2 superfamily, p53-deficient cells may be rendered chemotherapy-resistant (3–5).

During normal differentiation of hematopoietic cells, the relative expression of Bcl-2 family of proteins is temporally and topographically regulated. For example, in the lymphoid lineage, BCL-2 expression is critical for the development of B and T lymphoid progenitors from hematopoietic stem cells (6). Early T-cells demonstrate high levels of expression of BCL-2 relative to BCL-XL at the CD4-CD8- double negative stage, but downregulate BCL-2 in favor of BCL-XL during the CD4+CD8+ double positive stage (7–11). The expression of BCL-2 increases as the differentiation of T-cells proceeds into single positive thymocytes (CD4+CD8- or CD4-CD8+) (7, 8) and memory T-cells maintain relatively high expression (12). On antigen binding, co-stimulation of the T-cell receptor with CD28 upregulates BCL-XL (13). Similarly, during B-cell maturation, high levels of BCL-2 are seen early on (pro-B stage), are markedly lower in intermediate stages of differentiation, and again are upregulated in the memory B-cell (14, 15).

BCL-2 was first discovered as a partner of IGH in the translocation of chromosomes 14:18, which is a hallmark of follicular lymphoma and plays a central role in oncogenesis. Seminal studies demonstrated that cell lines that have upregulated anti-apoptosis protein like BCL-2 or MCL-1 do not themselves become cancerous but allow otherwise lethal mutations in the cell cycle to be transmitted to daughter cells. Additionally, upregulation of these inhibitory proteins by various means in a wide variety of tumor subtypes contributes to their resistance to chemotherapy (4). Taken together, the central role of the Bcl-2 superfamily in the development and progression of cancer, as well as in mediating chemotherapy resistance, has made it a highly sought-after therapeutic target.

## Pre-clinical Development of BH3 Mimetics

The targeting of BCL-2 proteins was initially evaluated using antisense mRNA compounds. Oblimersen is a BCL-2 antisense oligonucleotide that is complementary to part of the BCL-2 mRNA coding region, allowing for binding of BCL-2 mRNA to inhibit BCL-2 protein translation. Preclinical studies utilizing CD34+ marrow cells, BCL-2-positive myeloid cell lines and AML cells demonstrated inhibition of BCL-2 expression, decreased cell survival, and decreased clonal proliferation in cultures treated with BCL-2 antisense, as well as synergistic killing of AML cells in the presence of chemotherapeutics (16).

The development of BH3 mimetics allowed for effective targeting of anti-apoptotic proteins in tumors that are dependent on BCL-2 or BCL-XL and opened up a new avenue for targeted therapeutics in hematologic malignancies. These molecules inhibit the Bcl-2 family of anti-apoptotic proteins by occupying their BH3 binding groove. These include the synthetic small molecule BH3 mimetics developed by researchers at Abbvie, which were discovered utilizing nuclear magnetic resonance (NMR) to identify fragments with BCL-XL binding properties (17).

GX15-070/obatoclax was amongst the first compounds described to modulate the activity of the pro-survival Bcl-2 family members via inhibition of BH3 peptide binding. It is a polypyrrole developed from a natural compounds screen, which functions as a non-selective pan-BCL-2 inhibitor, binding BCL-2, BCL-XL, BCL-W, MCL1, among others. *In vitro* studies of obatoclax demonstrated antileukemic activity against AML cell lines and primary AML samples, via apoptosis and decreased proliferation (18). Mechanistically, its pro-apoptotic activity was demonstrated to involve release of Bim and Bak from BCL-2 and MCL1 with the formation of an active Bak/Bax complex. GX15-070/obatoclax has also been shown to overcome MCL1 mediated resistance to apoptosis (19).

ABT-737 was the first of the synthetic small molecule BH3 mimetics that was demonstrated to exhibit high potency inhibition of BCL-2, BCL-XL, and BCL-W, with greater affinity binding of these proteins by two to three orders of magnitude, compared to prior compounds (20). Notably, this molecule has been very useful in demonstrating the anti-tumor effect of BH3 mimetics in preclinical studies; however, solubility issues have prevented its use in the clinical setting. Specifically, regarding its activity in acute leukemias, ABT-737 was shown to induce apoptosis of AML cell lines, primary AML blast cells and stem cells, and in a murine xenograft model *in vivo* (21). In this study, overexpression of MCL-1 was shown to diminish the activity of GX15-070/obatoclax, highlighting a resistance mechanism to BCL-2/BCL-XL inhibition induced apoptosis. Further work demonstrated that ABT-737 induces the activation of extracellular receptor activated kinases (MAPK/ERK Kinases), resulting in upregulation of MCL-1 in AML cells (22). In this study, use of MEK inhibitors abrogated this effect of ABT-737, and combination treatment with ABT-737 and a MEK inhibitor resulted in potent synergistic killing of AML cell lines, primary AML blasts, and murine xenografts (22). ABT-737 additionally has been shown to have a potent synergistic effect in inducing killing of primary AML and MDS cells when used in combination with the hypomethylating agent azacitidine (23, 24). Treatment of primary AML cells with azacitidine has been shown to reduce MCL-1 expression, which likely accounts for this synergy (25). Of note, combination treatment with a FLT3 inhibitor in AML primary cells with an activating FLT3 mutation also demonstrated a synergistic effect (26). ABT-737 has further been shown to have activity in pediatric ALL, synergistically enhancing the effect of vincristine, dexamethasone, L-asparaginase in ALL cell lines and mice bearing ALL xenografts (27).

ABT-263/navitoclax, was subsequently developed via modification of ABT-737 at 3 molecular positions, resulting in oral bioavailability allowing for therapeutic use, while maintaining high potency inhibition of BCL-2, BCL-XL, and BCL-W (28, 29). In preclinical studies, navitoclax was demonstrated to have significant activity *in vitro* against ALL cell lines, as well as in ALL xenograft models (30). However, it was noted to have lower activity against CLL cells compared to its predecessor ABT-737, likely due to increased albumin binding (31). Additionally, its clinical use was limited by dose-dependent thrombocytopenia (32–34), due to on-target inhibition of BCL-XL, with resulting platelet apoptosis as well as impaired hemostatic functions of residual platelets (35, 36).

ABT-199/venetoclax is a modified BH3-mimetic which was subsequently designed to be highly selective for BCL-2 but not bind BCL-XL, as opposed to its predecessors. This specificity of venetoclax has been shown both *in vitro* and *in vivo* to result in a highly potent anti-tumor effect in BCL-2 dependent malignancies, while also sparing platelets. This specificity makes it a particularly attractive therapeutic candidate for acute leukemias, where thrombocytopenia is generally a significant complication of the disease, even prior to treatment. Pre-clinical studies have shown significant anti-tumor activity of venetoclax in acute leukemias. Using AML cell lines, murine AML xenografts, and primary patient AML myeloblasts Pan et al. demonstrated rapid venetoclax-induced apoptosis of AML cells both *in vitro* and *in vivo* (37). This study also described a novel method of determining a cell's relative dependence on anti-apoptotic proteins for survival, called BH3 profiling. In this method, exogenous sensitizers and activators are added to a cell and the degree of MOMP is determined. Using the unique binding patterns of the sensitizing and activating BH3 proteins, the group determined which anti-apoptotic proteins a cell was reliant on for continued survival. For instance, mitochondria that depolarize in the presence of exogenous HKR, which is known to exclusively bind and repress BCL-XL activity, would be said to be BCL-XL dependent. Using this technique, the group found a tight correlation between samples that were BCL-2 dependent and their sensitivity to ABT-199, suggesting on-target activity and indicating a potential biomarker for further exploration.

While the inhibition of BCL-2 by venetoclax results in significant anti-leukemic activity, overexpression of MCL-1 can lead to venetoclax resistance and has MCL-1 protein overexpression has been shown in venetoclax-resistant cells. Therefore, there is strong interest in combination therapy with drugs that overcome this mechanism of resistance. ABT-199 treatment of AML cells increases levels of MCL-1 protein, with resultant MCL-1 sequestration of the pro-apoptotic protein Bim, preventing the induction of apoptosis (38). Co-treatment of AML cells with venetoclax and either daunorubicin or cytarabine overcomes this resistance by increasing DNA damage, which in turn prevents MCL-1 upregulation (38). As with ABT-737, venetoclax in combination with azacitidine results in a synergistic anti-leukemic effect via azacitidine suppression of MCL-1. The venetoclax/azacitidine combination showed less potency against AML cell lines *in vitro* compared to ABT-737, but similar potency against primary AML and MDS samples tested *ex vivo* (23, 24). Combined treatment with venetoclax and the selective MCL-1 inhibitor A-1210477 abrogates MCL-1 sequestration of Bim and results in synergistically induced apoptosis in AML cell lines and primary patient samples (39). Bypassing the MCL-1 resistance mechanism has also been investigated with the pan-histone deacetylase inhibitor panobinostat, which was demonstrated to upregulate Bim, resulting in sustained Bim levels in the presence of venetoclax and a synergistic anti-leukemia effect in AML cell lines and patient samples (40).

The combinations of venetoclax and a variety of newer cancer therapeutics have additionally been investigated in preclinical studies in AML with promising results. Venetoclax and the cyclin-dependent kinase (CDK) inhibitor alvocidib were found to act synergistically in venetoclax sensitive and resistant AML models, with alvocidib acting to decrease MCL-1 and increase pro-apoptotic proteins Bim and NOXA, thereby complementing the venetoclax mechanism of action (41). Pevonedistat (MLN4924), an inhibitor of the Nedd8 activating enzyme, has also been shown to synergize with venetoclax in AML cell lines and in fresh AML patients' cells, through neutralization of MCL-1 and activation of the pro-apoptotic proteins Bax and Bak (42). Cotreatment of primary AML blasts and AML cell line xenografts with venetoclax and the PI3 kinase/mTOR inhibitor GDC-0980 also resulted in enhanced apoptosis, involving MCL-1 downregulation and Bax activation, suggesting that coadministration of PI3K and BCL-2 inhibitors may be effective in AML treatment (43).

The anti-leukemic effect of venetoclax in combination with the MDM2 antagonist idasanutlin has also recently been investigated. MDM2 is a negative regulator of the p53 tumor suppressor, and disruption of MDM2-p53 binding by idasanutlin allows for p53 activation and restores p53-dependent cell cycle arrest and apoptosis (44). Therefore, it was hypothesized that concomitant inhibition of BCL-2 and MDM2 would allow for an enhanced anti-leukemic effect by restoring key apoptotic and tumor suppressor pathways simultaneously. Indeed, the combination of venetoclax and idasanutlin results in enhanced anti-tumor activity compared to either treatment alone in p53 wild type AML cell lines *in vitro* and AML xenograft models *in vivo* (45). Further, combining these therapeutics was shown to overcome resistance to either drug alone, with p53 activation promoting MCL-1 degradation and BCL-2 inhibition shifting the p53 activation response from G1 cell cycle arrest to apoptosis, resulting in prolonged survival in murine xenograft models of resistant AML (46).

Overall, these studies suggest that the therapeutic effect of venetoclax in AML can be optimized in combination with other therapies, particularly those targeting leukemic cell mechanisms of venetoclax resistance. Interestingly, isocitrate dehydrogenase (IDH) 1 and 2 mutation status may also affect the response to BCL-2 inhibition, as IDH1 and IDH2 mutant primary human AML cells have been demonstrated to be more sensitive to venetoclax treatment than those with wild type IDH 1/2 (47). In this study, the mechanism of increased sensitivity to venetoclax was demonstrated to be via production of the oncometabolite R-2-hydroxyglutarate (R-2HG) by mutant IDH 1/2, with R-2HG mediated inhibition of cytochrome c oxidase activity in the mitochondrial electron transport chain, resulting in a lower mitochondrial threshold to trigger apoptosis under BCL-2 inhibition.

When considering the potential use of BH3 mimetics in MDS, blockade of BCL-2 with either venetoclax or ABT-737 has been demonstrated to result in significant toxicity to primary bone marrow cells from patients with high risk MDS or secondary AML, as demonstrated by reduction in CD34+ cells and decreased colony-forming capacity *in vitro*, but has no effect on bone marrow cells from patients with low risk MDS or healthy controls (48). This suggests that venetoclax treatment can overcome apoptotic resistance of high risk MDS cells and possibly delay disease progression in this patient population.

Regarding the potential of venetoclax use in ALL treatment, it has also been shown to have activity against T-cell ALLs in preclinical studies utilizing childhood ALL xenografts. Notably, the sensitivity of T cell ALLs to venetoclax appears to be dependent on the subtype of ALL and more specifically on the maturation stage of the leukemia cells (49, 50). Early T-cell precursors highly express BCL-2 and, correspondingly, ALL with an early T-cell progenitor phenotype show sensitivity to venetoclax. However, more mature subgroups of ALL are dependent on BCL-XL and not BCL-2, and, consequently, show sensitivity to navitoclax but not to venetoclax (49). An exception is mixed lineage leukemia (MLL) rearranged ALL, where venetoclax has been shown to recapitulate the activity of navitoclax (51).

Since MCL-1 overexpression allows for resistance to the BCL-2 inhibitors, the development of compounds that directly target MCL-1 is also of significant interest. Developing such compounds has proved challenging due to the shallower, less flexible binding site of MCL-1; however, direct MCL-1 inhibitors have recently been described (52). Amongst these is marinopyrrole A/maritoclax, a natural product identified from marine streptomycetes which binds MCL-1, but not BCL-XL, and disrupts the MCL-1-Bim interaction and induces proteosomal degradation of MCL-1 (53). *In vitro* studies have shown that maritoclax has an additional MCL-1-independent pro-apoptotic mechanism involving accumulation of mitochondrial reactive oxygen species (54). Maritoclax was demonstrated to induce apoptosis of AML cell lines and primary AML cells with elevated MCL-1 levels via MCL-1 downregulation and synergized with ABT-737 to overcome MCL-1-mediated ABT-737 resistance (55).

As with the small molecule BH3 mimetics, NMR-based fragment screen was used to identify compounds that inhibit MCL-1, including S63845 (and its derivative S64315) and A1201477. Intriguingly, these compounds cause increased MCL-1 protein levels, but also effectively disrupt the MCL-1-Bim interaction to induce apoptosis. S63845 was demonstrated to induce potent killing of MCL-1-dependent cancer cells, including leukemia and lymphoma cells, by activation of the BAX/BAK-dependent apoptotic pathway (56). A1201477 additionally was shown to induce synergistic effects with navitoclax in a variety of cancer cell lines (57).

## Clinical Development

### Oblimersen, BCL-2 Antisense

Anti-sense BCL-2, oblimersen sodium, was investigated early on in the development of therapy targeting this anti-apoptotic family. It was administered as a continuous infusion and showed clinical activity in patients with FLAG chemotherapy (fludarabine, cytarabine, and granulocyte stimulating factor) with relapsed or refractory AML or ALL (58) and with cytarabine and daunorubicin in untreated AML (59). Moore et al. further demonstrated clinical activity with 25% of patients achieving a response (60). Further, studies were halted, as oblimersen was limited by its continuous infusion administration and the lack of follow-up trials demonstrating clinical effectiveness.

### Obatoclax, Pan-BCL-2 Antagonist

Schimmer et al. investigated obatoclax, the pan-BCL-2 antagonist, in patients with AML, MDS, CLL, and ALL (61). Patients in this study were either ineligible for induction chemotherapy or had relapsed disease. Obatoclax was well tolerated in the study population. The most common adverse events (AE) were CNS-related, including somnolence, dizziness, fatigue, euphoric mood, and gait disturbance immediately following infusion; these resolved within a few hours after infusion administration. Complete remission was seen in one of 25 patients with AML, and three of 14 patients with MDS showed improvement of transfusion dependence. Arellano et al. investigated obatoclax use in patients with untreated myelodysplastic syndromes with anemia or thrombocytopenia (62). Dosing of 60 mg every 2 weeks seemed tolerable, with AE of euphoric mood, nausea, vomiting, grade 3-4 AE of anemia, thrombocytopenia, and pneumonia. This study was terminated as the response rate of 8% was below the pre-determined threshold for continuation.

### BCL-2 Inhibitors

#### Clinical Trials in AML

The initial clinical trials of the BCL-2 inhibitor venetoclax in AML were focused on dose finding, in light of the uncertain risk/benefit profile associated with tumor lysis syndrome (TLS), as well as on obtaining a preliminary assessment of the clinical efficacy of the drug. In a phase II study, Konopleva et al. evaluated venetoclax monotherapy at a dose of 800 mg in individuals with relapsed/refractory AML, or those unfit for intensive chemotherapy (63). In this study venetoclax had a favorable side effect profile as well as measurable clinical activity, with a 19% overall response rate (ORR). Common adverse events were nausea, vomiting, diarrhea, as well as febrile neutropenia and hypokalemia. Importantly, no TLS was observed in significant contrast to CLL where life-threating TLS has been seen.

In consideration of treatment of older patients with AML, Lin et al. designed a phase I study investigating venetoclax use with low-dose cytarabine in treatment-naïve patients aged >65 years with AML (64). Preliminary results of the study found an acceptable safety profile, with febrile neutropenia as the most prevalent adverse event. Tumor lysis syndrome (TLS) was not observed. Venetoclax dosing of 600 and 800 mg were assessed in the phase 1 portion of the study, with findings supporting 600 mg dosing moving forward in phase 2. Preliminary study findings showed promising clinical activity in this patient population, with an overall response rate of 44% in the phase I cohort. This clinical trial is ongoing with expected completion date of May 2019 (NCT02287233).

A further phase I study in newly diagnosed AML patients older than 65 years has been conducted by DiNardo et al., evaluating the efficacy of venetoclax in combination with the hypomethylating agents azacitidine or decitabine (65). Venetoclax doses of 400, 800, and 1,200 mg were assessed in this study, with doses of 400 and 800 mg utilized in the expansion, and patients were divided into subgroups based on the venetoclax dose and the hypomethylating agent received. Findings from this study have recently been published with impressive results and have led to accelerated FDA approval of venetoclax in this clinical setting. Study results include data analysis of 145 patients with a median age of 74 years and a median time on study of 8.9 months (66). Poor-risk cytogenetics were present in 49% of the study population. In the intent-to-treat population (including all doses of venetoclax), the rates of complete response (CR) and complete response with incomplete count recovery (CRi) were 37 and 30%, respectively, with an ORR (CR + CRi + partial response) of 68%. Evaluation of defined cohorts demonstrated CR + CRi rates of 73% for those who received 400 mg venetoclax and 65% for those who received 800 mg venetoclax. Of those patients who received 400 mg venetoclax + a hypomethylating agent, the response rates were similar between azacytidine and decitabine cohorts (76 and 71%, respectively). The median duration of CR + CRi was 11.3 months for all patients and 12.5 months for the 400 mg venetoclax group. The median overall survival was 17.5 months for all patients and had not been reached for the 400 mg venetoclax cohort. Further, the authors evaluated minimal residual disease (MRD) status and found that amongst those who achieved CR/CRi, 29% (28/97) had at least 1 assessment of MRD <10^−3^, and of those with MRD negativity, the median duration of response and overall survival were not reached. The most common toxicities were gastrointestinal and hematologic in nature, noting that many hematologic adverse events were attributable to the underlying hematologic disease, and the most common grade 3 and 4 adverse events included febrile neutropenia, cytopenias, and pneumonia. Notably, subgroup analyses also demonstrated meaningful responses in patients with poor risk cytogenetics. In particular, those with NPM1 mutations had a quite favorable outcome with venetoclax-based therapy, with CR + CRi rate of 91.5% and median overall survival and duration of CR + CRi not reached. NPM1 mutation status was additionally found to be a statistically significant predictor of favorable outcome. Patients harboring FLT3 and IDH1/2 mutations also demonstrated impressive responses, with CR + CRi rates of 72 and 71%, respectively.

Looking ahead, results of a phase 3 randomized, double-blind, placebo-controlled study investigating venetoclax use in older AML patients are anticipated to be favorable (67). The study is designed to evaluate the effect of venetoclax (at 400 mg dose) in combination with azacitidine on overall survival and remission rates as compared to the combination of azacitidine with placebo. Secondary outcomes include event free survival and quality life measures. The study opened in February 2017, with a target enrollment of 400 patients and an estimated completion date of the trial is January 2021 (NCT02993523), though given the FDA approval of this medication, continued accrual may be slow.

The use of venetoclax in combination with other agents for the treatment of AML is also being investigated. DiNardo and colleagues recently reported findings of various venetoclax combinations in relapsed and refractory AML, MDS, and blastic plasmacytoid dendritic cell neoplasm (BPDCN) (68). A total of 43 patients with median age 68 years (range 25–83) were evaluated, the majority of whom had already received more than 2 s line regimens. The combination regimens included decitabine, azacitidine, low-dose cytarabine, fludarabine plus cytarabine, and cladribine plus cytarabine. Midostaurin, sorafenib, and ruxolitinib were also used in the few patients who had -FLT3-ITD or JAK2-mutations. Twenty-one percent of patients had complete response, complete remission with incomplete blood count recovery, or morphologic leukemia-free state. Interestingly, they also found a 27% response rate amongst patients with IDH-1 or IDH-2 mutations (11 patients total) when an IDH inhibitor was used in combination with venetoclax, despite previous non-responsiveness to an IDH-1 or IDH-2 inhibitor. The authors concluded that low intensity chemotherapy in combination with venetoclax is an option for therapy in multiply relapsed/refractory AML, MDS, and BPDCN. DiNardo et al. are also currently investigating the safety and tolerability of venetoclax when utilized in induction, consolidation, and maintenance of newly diagnosed or refractory AML together with standard intensive care chemotherapy with fludarabine, cytarabine, idarubicin, filgrastim (FLAG-IDA). The study is actively recruiting, and its completion date is estimated for September 2024 (NCT03214562). Additionally, this group of researchers will be investigating the safety and tolerability as well as clinical efficacy of venetoclax used with ivosidenib (AG120) in IDH-1 relapsed or refractory AML. The study opened in March 2018 and is actively enrolling with an estimated completion date of September 2020 (NCT03471260).

#### Clinical Trials in ALL

An ongoing phase I, open-label, multicenter trial is investigating venetoclax and navitoclax use with standard chemotherapy regimen in young adults and pediatric patients with T-cell and B-cell ALL (69). Serious adverse events observed in this trial are comparable to other studies, and include nausea, vomiting, abdominal pain, somnolence, and pseudomonas sepsis. Preliminary data reveal all four of the enrolled patients achieved a clinical response, either complete remission, complete remission with incomplete platelet recovery, or complete remission with incomplete marrow recovery. One patient had disease progression at 3.4 months and discontinued trial therapy. Estimated completion date of trial is June, 2020 (NCT03181126). This study, in conjunction with NCT03236857, which is investigating venetoclax monotherapy followed by chemotherapy regimens (70), will help further elucidate the clinical utility of targeting this anti-apoptotic family in pediatric and adult ALL.

Palmisiano and colleagues initiated an ECOG-ACRIN, multicenter phase Ib/II study (EA9152) investigating the combination of venetoclax and liposomal vincristine in relapsed/refractory or unresponsive to treatment childhood and adult B-cell and T-cell ALL (NCT03504644). This study will seek to elucidate the optimal dose of venetoclax in this combination regimen in patients with relapsed/refractory T/B-ALL by establishing the maximum tolerated dose (MTD), as well as assessing the preliminary efficacy of the combination, using response rate as a primary endpoint. This trial started April, 2018 and is currently enrolling, with expected completion date April, 2021.

#### Clinical Trials in MDS

Data demonstrating efficacy of BCL-2 inhibition in MDS is largely preclinical at this point. As noted above, DiNardo et al. did include patients with MDS in addition to those with AML in their recent report of 43 patients treated with venetoclax combination therapy for relapsed/refractory disease (68). However, amongst those 43 patients, only 2 carried a diagnosis of MDS and only 1 one of these patients responded. The patient who responded notably had MDS with complex cytogenetics and mutations of RUNX1 and TP53, and had been extensively pretreated, including prior azacitidine and decitabine as well as two prior allogeneic haplo-identical stem cell transplants. However, despite the paucity of clinical data of BCL-2 inhibitors in MDS, there are current clinical trials evaluating venetoclax in MDS which are actively recruiting. The two trials specifically limited to those with patients with MDS are phase 1b studies sponsored by AbbVie. These include evaluation of venetoclax in combination with azacitidine in treatment-naïve higher-risk MDS patients (NCT02942290) and evaluation of venetoclax alone or with azacitidine in relapsed/refractory MDS patients (NCT02966782). Both studies opened in early 2017, with estimated primary completion dates of September 2019 and August 2020, respectively.

## MCL-1 Inhibitors

Currently, there are trials involving MCL-1 inhibitor use in AML/MDS ongoing as well, with initial aims investigating tolerated doses and adverse effects of these intravenous agents. The first in human trial of the MCL-1 inhibitor AMG176 (Amgen) in patients with relapsed/refractory multiple myeloma and AML is currently active (NCT02675452). The trial start date was June 2016 with an expected primary completion date of May, 2020. The primary objective of this phase I trial is to investigate the safety profile and maximum tolerated dose of AMG176 in patients with relapsed/refractory AML and multiple myeloma, with a secondary objective to evaluate preliminary efficacy of the therapeutic agent. A second study investigating another MCL-1 inhibitor, S64315 (Institut de Recherches Internationales Servier) in patients with AML or MDS is also currently active (NCT02979366). This phase 1 open-label, non-randomized, non-comparative study will investigate the safety, tolerability, and incidence of dose-limiting toxicities of the drug. The study started March 2017 with estimated date of completion of October 2020.

## Conclusions

The ability of cancers to upregulate the Bcl-2 family of anti-apoptotic proteins as a means to evade apoptosis represents a key mechanism for treatment resistance and relapse in acute leukemia and MDS. Targeted inhibition of the anti-apoptotic Bcl-2 proteins has been shown to have impressive efficacy in the treatment of several hematologic malignancies, with current FDA approvals of venetoclax for the treatment of CLL and AML. Preclinical studies have demonstrated substantial anti-leukemic activity of the BH3 mimetics, and there are many ongoing trials to confirm these findings. Given its bioavailability and tolerability, venetoclax is the main BH3 mimetic in these current clinical investigations (see Table 1). While these studies are ongoing, many questions remain. The anti-leukemic activity of the BH3 mimetics is limited by drug resistance mechanisms that are gradually being characterized in pre-clinical models, *in vitro* and *in vivo*, and while the primary mechanisms of resistance *in vivo* remain unclear, clinical studies thus suggest that combination strategies are able to overcome resistance and lead to clinical responses. Determining which combination therapies provide optimal clinical efficacy is a very important goal, moving forward, and further studies are needed to explore novel combination strategies. The role of venetoclax and other BH3 mimetics in standard induction and consolidation therapy is also of great interest. Further, determining which subgroups of patients are likely to derive the most clinical benefit from BH3 mimetics would be quite impactful clinically, and the use of BH3 profiling and the identification of other potential biomarkers in leukemia and MDS patients to guide the therapeutic application of these drugs requires further investigation. Despite these uncertainties, BCL-2 inhibition with venetoclax, and MCL-1 or BCL-XL inhibition potentially, represent a significant advance in targeted treatment approaches to hematologic malignancies and are very promising in acute leukemia and MDS treatment.

**Table 1:** Active clinical trials of therapy targeting BCL-2 family in AML, MDS, ALL.

**NCT Identified**	**Investigated Drug**		**Monotherapy/Combination**		**Disease**	**Stage**	**Phase**	**Recruitment**	**Primary investigators**	**Estimated date of completion**
NCT03214562	Venetoclax	Bcl-2 Inhibitor	Combination	Fludarabine, cytarabine, Idarubicin, filgrastim, pegylated filgrastim	AML	Newly diagnosed; Relapsed/Refractory	I/II	Recruiting	Courtney DiNardo, MD	Sep-18
NCT03471260	Venetoclax	Bcl-2 Inhibitor	Combination	Ivosidenib	AML	Relapsed/Refractory	I/II	Recruiting	Courtney DiNardo, MD	Sep-20
NCT02203773	Venetoclax	Bcl-2 Inhibitor	Combination	Azacitidine, decitabine	AML	Newly diagnosed AML	I/II	Active, not Recruiting	Courtney DiNardo, MD	Mar-19
NCT02920541	S 055746	Bcl-2 Inhibitor	Monotherapy		AML,MDS	Newly diagnosis; Relapsed/Refractory	I	Active, Not Recruiting	Andrew Wei, MBBS, PhD	Aug-18
NCT02993523	Venetoclax	Bcl-2 inhibitor	Combination	Azacitadine	AML	Newly diagnosed	III	Recruiting	AbbVie Inc.	May-20
NCT02287233	Venetoclax	Bcl-2 Inhibitor	Combination	Cytarabine	AML	Newly diagnosed	I/II	Active, not recruiting	AbbVie Inc.	May-19
NCT03181126	Venetoclax and Navitoclax	Bcl-2 Inhibitor and Bcl-2, Bcl-Xl, and Bcl-Winhibitor	Combination	peg-asparaginase (+vincristine + dexamethasone + tyrosine kinase inhibitor (TKI) (if applicable)	ALL, lymphoblastic leukemia	Relapsed/Refractory	I	Recruiting	AbbVie Inc.	Jun-20
NCT03236857	Venetoclax	Bcl-2 Inhibitor	Combination	Various	ALL, AML, NHL, neuroblastoma	Relapsed/Refractory	I	Recruiting	AbbVie Inc.	Apr-22
NCT02979366	S 64315	Mcl-1 Inhibitor	Monotherapy		AML, MDS	Relapsed/Refractory; Secondary MDS; Unfit, Elderly AML	I	Recruiting	Institut de Recherches Internationales Servier Clinical Studies Department	Oct-20
NCT02675452	AMG 176	Mcl-1 Inhibitor	Monotherapy		AML, MDS	Relapsed/Refractory	I	Recruiting	Amgen	Oct-18
NCT03504644	Venetoclax	Bcl-2 Inhibitor	Combination	Vincristine Liposomal	T-cell or B-Cell ALL	Relapsed/Refractory	Ib/II	Recruiting	Neil Palmisiano, MD	Apr-21
NCT03466294	Venetoclax	Bcl-2 Inhibitor	Combination	Azacitadine	AML	Newly diagnosed, elderly, treatment naïve	II	Recruiting	Daniel Pollyea, MD	Jun-21
NCT03484520	Venetoclax	Bcl-2 Inhibitor	Combination	Dinaciclib (MK7965)	AML	Relapsed/Refractory	I	Recruiting	AbbVie	Jun-21
NCT03404193	Venetoclax	Bcl-2 Inhibitor	Combination	Decitabine	AML,MDS	Relapsed/Refractory; High-risk	II	Recruiting	Marina Konopleva, MD	Dec-20
NCT03613532	Venetoclax	Bcl-2 Inhibitor	Combination	Fludarabine, busulfan	AML, MDS, MDS/	AML, MDS, CMML, MDSprior to transplant	I	Not yet recruiting	Jacqueline S. Garcia, MD	Feb-21
NCT03573024	Venetoclax	Bcl-2 Inhibitor	Combination	Azacitidine	Non-elderly AML	Treatment naïve	II	Not yet recruiting	Daniel Pollyea, MD	Jun-23
NCT03625505	Venetoclax	Bcl-2 Inhibitor	Combination	Gliteritinib	AML	Relapsed/Refractory	I	Not yet recruiting	AbbVie Inc.	Jul-21
NCT03441555	Venetoclax	Bcl-2 Inhibitor	Combination	Alvocidib	AML	Relapsed/Refractory	I	Recruiting	AbbVie Inc.	May-21
NCT03455504	Venetoclax	Bcl-2 Inhibitor	Combination	Fludarabine, Cyratabine and Idarubicine	AML	New onset, non-M3 AML	II	Not yet recruiting	Fabio Ciceri, MD	Jun-20
NCT02942290	Venetoclax	Bcl-2 Inhibitor	Combination	Azacitidine	MDS	Treatment naïve, High- risk	I	Recruiting	AbbVie Inc.	Dec-20
NCT02966782	Venetoclax	Bcl-2 Inhibitor	Monotherapy/Combination	Azacitidine	MDS	Relapsed/Refractory	I	Recruiting	AbbVie Inc.	Aug-20
NCT03069352	Venetoclax	Bcl-2 Inhibitor	Combination	Low dose cytarabine	AML	Treatment naïve, Ineligible for chemotherapy	III	Recruiting	AbbVie Inc.	Nov-19
NCT03629171	Venetoclax	Bcl-2 Inhibitor	Combination	Liposome encapsulated Daunorubicin-Cytarabine	AML	Treatment naïve; Relapsed/refractory	II	Not yet recruiting	Tapan Kadia, MD	Jun-20
NCT03576547	Venetoclax	Bcl-2 Inhibitor	Combination	Ponatinib	ALL	Relapsed/Refractory	I/II	Recruiting	Farhad Ravandi-Kashani, MD	Jan-19
NCT02670044	Venetoclax	Bcl-2 Inhibitor	Combination	Cobimetinib orIdasanutlin	AML	Relapsed/Refractory; Ineligible for chemotherapy	I/II	Recruiting	Hoffmann-La Roche	Jan-20
NCT03194932	Venetoclax	Bcl-2 Inhibitor	Combination	Cytarabine, Idarubicin, intrathecal Triple Therapy	AML	Pediatric Relapsed/Refractory or Ambiguous Lineage	I	Recruiting	Jeffrey E Rubnitz, MD, PhD	Aug-20
NCT03586609	Venetoclax	Bcl-2 Inhibitor	Combination	Cladribine, Low-dose Cytarabine, Azacitidine	AML	Treatment naïve	II	Not yet recruiting	Tapan Kadia, MD	Jan-21

## Author Contributions

AM, SH, LW, CV, CE, MK, and NP provided substantial contribution to the conception, drafting, editing, and final approval of this manuscript.

### Conflict of Interest Statement

CE and NP receive research funding from AbbVie. The remaining authors declare that the research was conducted in the absence of any commercial or financial relationships that could be construed as a potential conflict of interest.
